# Supplementary material for: Novel Driver Strength Index highlights important cancer genes in TCGA PanCanAtlas patients
Source: PeerJ. 2022 Aug 11;10:e13860. doi: 10.7717/peerj.13860 (PMC9375969; doi:10.7717/peerj.13860)
Supplement: Supplemental Information 2 [file peerj-10-13860-s002.zip › COHORTS/genes plots/age/2021_8_16_14_9_distribution_cohorts_females_30-34.pdf]

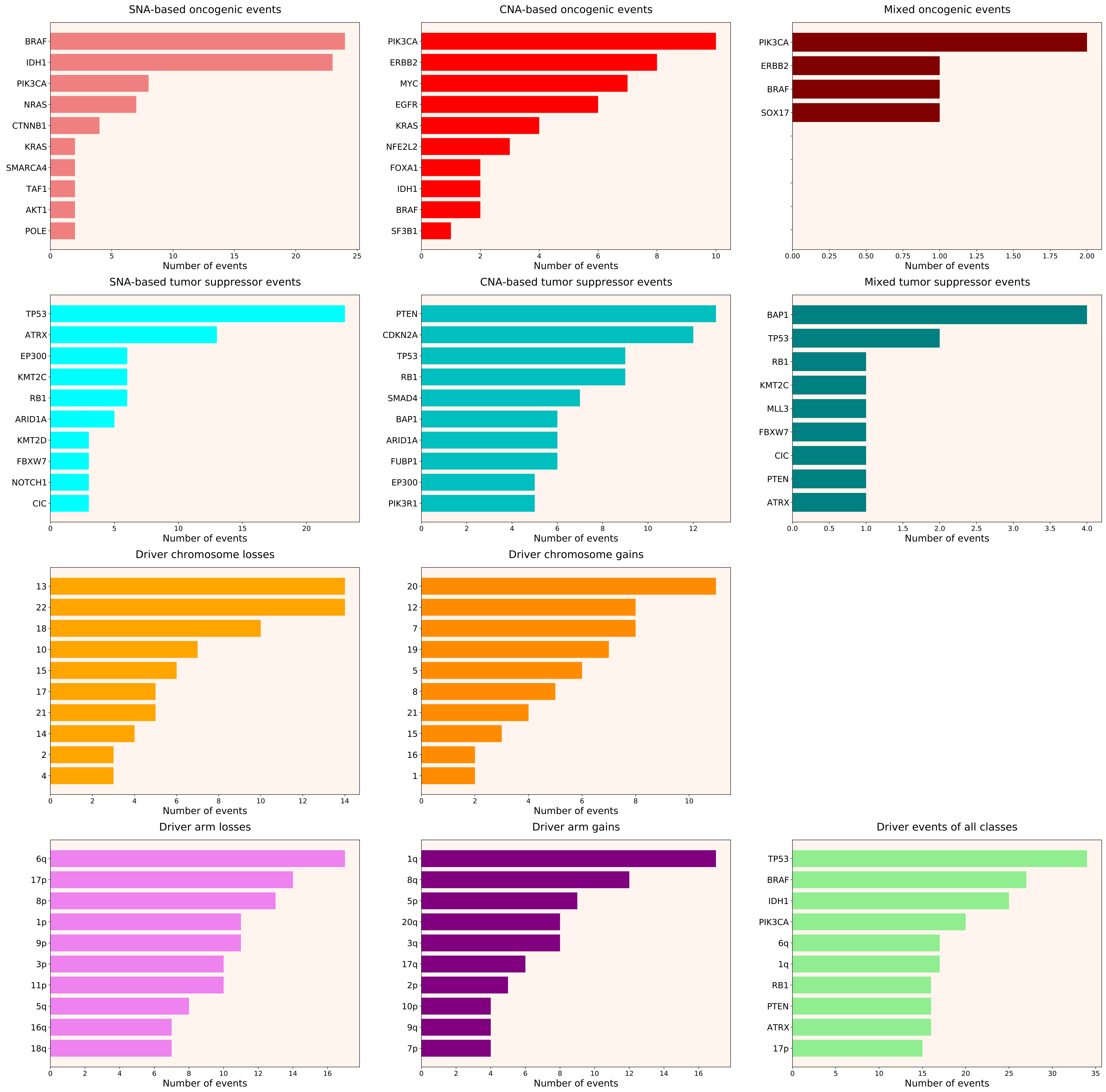

SNA-based oncogenic events
BRAF
IDH1
PIK3CA
NRAS
CTNNB1
KRAS
SMARCA4
TAF1
AKT1
POLE
Number of events
CNA-based oncogenic events
PIK3CA
ERBB2
MYC
EGFR
KRAS
NFE2L2
FOXA1
IDH1
BRAF
SF3B1
Number of events
Mixed oncogenic events
PIK3CA
ERBB2
BRAF
SOX17
Number of events
SNA-based tumor suppressor events
TP53
ATRX
EP300
KMT2C
RB1
ARID1A
KMT2D
FBXW7
NOTCH1
CIC
Number of events
CNA-based tumor suppressor events
PTEN
CDKN2A
TP53
RB1
SMAD4
BAP1
ARID1A
FUBP1
EP300
PIK3R1
Number of events
Mixed tumor suppressor events
BAP1
TP53
RB1
KMT2C
MLL3
FBXW7
CIC
PTEN
ATRX
Number of events
Driver chromosome losses
13
22
18
10
15
17
21
14
2
4
Number of events
Driver chromosome gains
20
12
7
19
5
8
21
15
16
1
Number of events
Driver arm losses
6q
17p
8p
1p
9p
3p
11p
5q
16q
18q
Number of events
Driver arm gains
1q
8q
5p
20q
3q
17q
2p
10p
9q
7p
Number of events
Driver events of all classes
TP53
BRAF
IDH1
PIK3CA
6q
1q
RB1
PTEN
ATRX
17p
Number of events
